# Evaluation of antimicrobial activity of the hydrolate of *Coridothymus capitatus* (L.) Reichenb. fil. (Lamiaceae) alone and in combination with antimicrobial agents

**DOI:** 10.1186/s12906-020-2877-x

**Published:** 2020-03-17

**Authors:** Andreana Marino, Antonia Nostro, Narcisa Mandras, Janira Roana, Giovanna Ginestra, Natalizia Miceli, Maria Fernanda Taviano, Fabrizio Gelmini, Giangiacomo Beretta, Vivian Tullio

**Affiliations:** 1 Department of Chemical, Biological, Pharmaceutical and Environmental Sciences, University of Messina, Polo Annunziata, 98168 Messina, Italy; 2 Department of Public Health and Pediatrics, Microbiology Division, University of Turin, Via Santena 9, 10126 Turin, Italy; 3 Department of Environmental Science and Policy – ESP, University of Milan, Via Celoria, 2, 20133 Milan, Italy

**Keywords:** *Coridothymus capitatus* (L.) Reichenb. Fil., Essential oil, Hydrolate, Antimicrobial, Synergism, Mechanism of action

## Abstract

**Background:**

Hydrolates, complex mixtures containing traces of essential oils (EOs), are inexpensive, easy to make and less toxic than their corresponding EOs. The antibacterial and antifungal activity of the hydrolate of *Coridothymus capitatus* (L.) Reichenb. fil. (Lamiaceae) alone and in combination with antimicrobial drugs, such as tetracycline and itraconazole, were evaluated.

**Methods:**

The chemical composition was analysed by gas-chromatography-mass spectrometry (GC-MS). Standard methods were performed to evaluate the susceptibility of some Gram-positive and Gram-negative bacteria, and *Candida* spp. to the hydrolate, in comparison with its EO. The hydrolate mechanism of action was assayed by propidium iodide and MitoTracker staining. Checkerboard tests were carried out for combinations studies.

**Results:**

GC-MS identified 0.14% (v/v) of total EO content into hydrolate and carvacrol as a dominant component. The hydrolate showed a good antimicrobial activity against bacteria and yeasts. It exhibited a synergistic effect with itraconazole against *Candida krusei*, and an additive effect with tetracycline against methicillin-resistant *Staphylococcus aureus* strains. Hydrolate changed the membranes permeability of bacteria and yeasts and altered mitochondrial function of yeasts.

**Conclusions:**

Our study extends the knowledge by exploiting non-conventional antimicrobial agents to fight the emergence of antibiotic resistance.

## Background

Essential oils (EOs) and aromatic oily compounds extracted from plant material, have been suggested as potential sources of new antimicrobial and therapeutic products [1–5]. In nature, EOs play a role in plant defence against microorganisms and insects [6]. In addition, they are valuable natural compounds used in many fields, i.e. pharmaceutical and food and cosmetic industries [7]. EOs composition is influenced by many factors such as species, cultivar, geographic and climatic conditions, cultivation practices, storage conditions of raw materials. Thus, wild plants of the same species due to different backgrounds can express many characters and chemical composition [8].

The genus *Thymus* L. (Lamiaceae) includes several species with biological properties, based on a number of active components such as carvacrol, thymol, p-cymene and γ-terpinene, known to exhibit different antimicrobial activity [9]. Medicinal plants of the genus *Thymus* are traditionally administered for whooping coughs, upper respiratory congestions, acute and chronic bronchitis, and gastrointestinal disorders [10].

*Coridothymus capitatus* (L.) Reichenb. fil. [syn. *Thymus capitatus* (L.) Hoffmanns. & Link], also known as Spanish origanum, is a Mediterranean aromatic shrub, extensively found all over Italy [11]. The EO, obtained from the flowers by hydrodistillation, is important for pharmaceutical and food and cosmetic industries. During distillation, parts of the EO components remain dissolved in the distillation water and the “product” is called hydrolate, which is also known as the aromatic water, floral water or hydrosol [12]. Hydrolates are complex mixtures containing traces of related EOs, and many water-soluble components. They are easy and inexpensive to produce and less toxic than their corresponding EOs [13, 14]. Hydrolates are used in the aqueous phase in the manufacture of lotions, and creams and soaps, or independently as tonics and air fresheners, as well as in the food industries. Since some previous evidence showed that hydrolates could have antimicrobial properties [15], in the present research we evaluated the antibacterial and antifungal activity of the hydrolate obtained from *C. capitatus* (L.) Reichenb. fil. grown wild on the sunny slopes and rocky crags of Ragusa, arid area of Sicily (Italy). Moreover, we studied the effect of hydrolate alone and in association with antimicrobial agents, such as tetracycline (TC) and itraconazole (ITC). TC is an inexpensive broad-spectrum antibiotic extensively used in the prophylaxis and therapy of bacterial diseases. However, the widespread of bacterial resistance caused to efflux and ribosomal protection mechanisms limited tetracycline’s effectiveness [16]. ITC is a lipophilic antifungal drug with lower toxicity, and may be used in association with EOs that are lipophilic compounds [17].

## Methods

### Plant material and extraction procedure

The hydrolate and EO of *C. capitatus* (L.) Reichenb. fil. (Batch: BI25A10102. Exp. 10/2017) were supplied by Exentiae S.R.L. (Catania, Sicily, Italy). They were obtained from plants grown in a classified area “Lauretum-Rosmarinetum”, of the Hyblaean Mountains, near Ragusa, Sicily. A voucher specimen was deposited in the Herbarium Mediterraneum Panormitanum housed in the Botanical Garden of Palermo, Italy (id number 71381). The hydrolate and EO were obtained by steam distillation in a Clevenger-type apparatus from dried flowers collected at the beginning of the flowering stage.

### Gas chromatography/mass spectrometry (GC-MS) analysis

The *C. capitatus* hydrolate and EO were analysed using a Bruker Scion SQ gas chromatograph (Bruker Daltonics, Macerata, Italy), coupled with a single quadrupole (SQ) detector. A Zebron ZB -5HT Inferno capillary column (VF-5 ms, 30 m × 0.25 mm i.d., film thickness 0.25 μm) was used for separation. GC ramp: 60 °C (hold time 3 min), 60 to 150 °C (3.0 °C/min, hold 1 min), 150–380 °C (10 °C/min, hold 3 min). Injector temperature: 250 °C, hold 20 min. Helium 5.5 was used as carrier gas and the column gas flow settled to 1.00 mL/min.; ionization energy − 70 eV. Split/splitless ratio 1:30 after 45 s. Peaks identification was performed by retention indices, evaluated using the homologue n-alkane scale and by comparison of the experimental mass spectra fragments with those of the NIST mass spectral database (vers. 2.0, 2011), as well as with those of commercial standards. The relative percentage of the components was obtained by normalization of the peaks area.

EO (batch: BI25A10102) was diluted 1:1000 (v/v) in Ethylacetate, and 1 μL injected in GC-MS. The hydrolate was diluted 1:5 (v/v) in EtOH, and 1 μL injected in GC-MS.

The EO percentage (m/m) content in the hydrolate was evaluated by extracting 3 mL of hydrolate with 9.0 mL of CH_2_Cl_2_ (*n* = 3) [18]. After separation, the organic phases were pooled and the solvent evaporated under reduced pressure (IKA HB 10 basic, 280 rpm, T = 40 °C), until constant weight. The carvacrol (Aldrich-Fluka-Sigma S.r.l., Milan, Italy) percentage was evaluated by comparison of the GC-MS peak area with a calibration curve (y = 118415306.12x - 1083867346.94, r2 = 0.99946, *n* = 5 points). Results were obtained from three independent experiments performed in triplicate.

### Antimicrobial agents

TC hydrochloride (superior quality) was purchased from Ningxia Qiyuan Pharmaceutical Co. LTD (No. I Qiyuan Street, Wangyyuan Industrial Area, Yinchuan, Ningxia, China) and ITC was obtained from Sigma-Aldrich (purity determined by HPLC = ≥98 percentage; n° I6657). TC stock solution was dissolved in phosphate buffered solution, pH 7 (PBS; Sigma-Aldrich), whereas ITC stock solutions in dimethylsulfoxide (100%; DMSO; Sigma-Aldrich), and then stored at − 20 °C.

### Bacteria

Bacteria included in this study were Gram-positive and Gram-negative strains obtained from the in-house culture collection of the Department of Chemical, Biological, Pharmaceutical and Environmental Sciences, University of Messina (Italy). The Gram-positive bacteria included reference strains: *Staphylococcus aureus* ATCC 6538*, S. aureus* ATCC 43300, *S. epidermidis* ATCC 35984; *Listeria monocytogenes* ATCC 13932; *Bacillus subtilis* ATCC 6633 and some clinical isolates: *S. aureus* 7786*, S. aureus* 815 (methicillin resistant *S. aureus*-MRSA) and *S. aureus* 74CCH-MRSA. The Gram-negative strain was *Pseudomonas aeruginosa* ATCC 9027 [19]. The identification of all isolates was conducted using API systems (bioMérieux, Firenze, Italy). Isolates were stored at − 70 °C in Microbanks™ vials (DID, Pro-Lab Diagnostics, Ontario, Canada), until use.

### Yeasts

The following yeast strains, obtained from the MiBat-TUCC collection of the Public Health and Pediatrics Department, Microbiology Section, Bacteriology and Mycology Laboratory, University of Turin (Italy), were tested: *Candida albicans* ATCC 90028, *C. albicans* ATCC 10231, *C. glabrata* ATCC 90030; clinical isolates of *C. albicans* 183, *C. krusei* 398, *C. glabrata* 32–09, *C. norvegensis* 112, *C. lusitaniae* 103, *C. valida* 287, *C. guilliermondii* 209, *C. parapsilosis* 198, and *C. tropicalis* 16–09. The clinical yeasts were collected from hospitalized patient specimens in Torino, identified by API systems (API ID32C panel), and stored at − 80 °C in Microbanks™ vials (DID), until use.

### Antibacterial susceptibility testing

Bacterial cultures for antibacterial activity assays were grown in Mueller-Hinton Broth (MHB, Oxoid, Basingstoke, United Kingdom) for 24 h at 37 °C. Working cultures of bacteria were adjusted to the required inoculum of 10^5^ CFU/mL. The Minimum Inhibitory Concentration (MIC) and the Minimum Bactericidal Concentration (MBC) of drugs and natural compounds were established by broth microdilution method, according to Clinical and Laboratory Standards Institute (CLSI, document M07-Ed11, 2018), with some modifications for hydrolate, and EO [20]. The hydrolate was used as such. The EO was dissolved to 5% using DMSO and further diluted using MHB to 2–0.016%. DMSO maximum concentration was 1% (v/v). Serial doubling dilutions of the hydrolate (100% product) and EO were prepared in a 96-well microtiter plates over the range of 50–0.1% (v/v) and 2–0.016% (v/v) in MHB, respectively. Growth controls (medium with inocula but without hydrolate or EO) were included. TC was tested against all strains at concentrations ranging from 32 to 0.016 μg/ml. Plates were incubated at 37 °C for 24 h. The MIC was considered as the lowest concentration of the hydrolate or the EO, at which there was no microbial growth. To determine MBC, bacterial aliquots (10 μL) were taken from each well and inoculated in Mueller-Hinton Agar (MHA, Oxoid). Cultures were incubated for 24 h at 37 °C. Results were obtained from three independent experiments performed in triplicate.

### Antifungal susceptibility testing

Yeast cultures for antifungal activity tests were grown at 30 °C (24 h) in RPMI-1640 (0.2% glucose) supplemented with L-glutamine (Sigma-Aldrich), and 0.165 M 3-(N-Morpholino) propanesulfonic acid (MOPS) (pH 7) (Sigma-Aldrich), without sodium bicarbonate. Working cultures of yeasts were adjusted to the required concentration of 10^3^ CFU/mL. The MIC and the Minimum Fungicidal Concentration (MFC) of drugs and natural compounds were detected by broth microdilution method, according to CLSI document M27-A3, 2008, with some modifications for hydrolate, and EO [21].

The hydrolate was used as such. The EO was dissolved to 5% using DMSO, and then diluted using RPMI-1640 plus MOPS to 2–0.016% (v/v). DMSO maximum concentration was 1% (v/v). Serial doubling dilutions of the hydrolate (100% product) and EO were prepared in a 96-well microtiter plates over the range of 50–0.1% (v/v) and 2–0.016% (v/v) in RPMI-1640 plus MOPS, respectively. Cultures were incubated for 24 h at 35 °C. Growth controls (medium with inocula but without hydrolate or EO) were included. MIC was considered the lowest concentration of hydrolate or the EO at which no microorganism growth was detected. MIC of ITC was defined as the lowest drug concentration that inhibited ≥50% growth inhibition in comparison with the control. To determine the MFC, fungal aliquots (10 μL) were taken from each well and spreaded onto Sabouraud Dextrose Agar (SDA). Cultures were incubated at 35 °C for 48 h [22, 23]. To define yeasts resistance to ITC published epidemiological cut-off values (ECVs) were used (ECV, 1 μg/mL) [24]. Results were obtained from three independent experiments performed in triplicate.

### Checkerboard test

Based on the antibacterial and antifungal susceptibility testing results, MRSA strains (*S. aureus* ATCC 43300, *S. aureus* 815, *S. aureus* 74CCH), and some yeasts strains (*C. albicans* ATCC 90028, *C. albicans* ATCC 10231, *C. albicans* 183, *C. glabrata* 32–09*,* and *C. krusei* 398) were used to evaluate the efficacy of hydrolate in combination with TC or ITC, respectively. The checkerboard assay was used to assess drug synergism [25]. The ranges of TC/hydrolate were based on the MIC of the two compounds. Bacterial suspensions were prepared in MHB to yield an inoculum of 5 × 10^5^ CFU/mL. The ranges of ITC/hydrolate were based on the MIC of the two compounds. Yeast suspensions were prepared in RPMI plus MOPS to yield an inoculum of 1.5 × 10^3^ CFU/mL. Microplates were read after 24-48 h at 37 °C (bacteria and yeasts). Data were interpreted by the fractional inhibitory concentration index (FICI). A FICI value ≤0.5 was referred to synergy, whereas values between 0.5 and 1.0 were considered as additive. FICI values > 4.0 were interpreted as antagonism and FICI values between 1.0 and 4.0 were considered as indifferent [25]. Results were obtained from three independent experiments performed in triplicate.

### Propidium iodide staining

To analyse the membrane integrity, fraction of surviving cells of *S. aureus* ATCC 6538 and *C. albicans* ATCC 10231 exposed to hydrolate were stained with propidium iodide (PI) solution (Sigma-Aldrich) [26]. The control sample of both strains was performed for comparison. Briefly, treated cells (0.5 MIC) were washed and suspended in PBS. Then, PI solution (final concentration of 1.25 μg/mL) was added to these cell suspensions and incubated for 10 min at 25 °C. After that, cells were washed twice with PBS to eliminate the excess of the stain and immediately examined under the inverted microscope Axio Observer.Z1 with ApoTome.2 (Zeiss, Milan, Italy). PI has a maximum emission peak at 606 nm. Results were obtained from three independent experiments performed in triplicate.

### MitoTracker staining

To detect permeability changes of mitochondrial membrane, fraction of surviving cells of *C. albicans* ATCC 10231 exposed to hydrolate was stained with mitochondrion-specific dye MitoTracker®RedCMXRos (MTR) according to manufacturer’s instructions (Invitrogen, Fisher Scientific Italia, Rodano-Milan, Italy). The control sample of the strain was performed for comparison. The treated cells (0.5 MIC) were washed with PBS by centrifugation, and stained with 50 nM MTR for 15 min at 35 °C [26–28]. Stained cells were rinsed twice with PBS and examined by the above-mentioned fluorescence microscope. MTR has a peak of excitation at 579 nm and a peak of emission at 599 nm. Results were obtained from three independent experiments performed in triplicate.

## Results

### Chemical analysis of *C. capitatus* essential oil and hydrolate

**Fig. 1:**
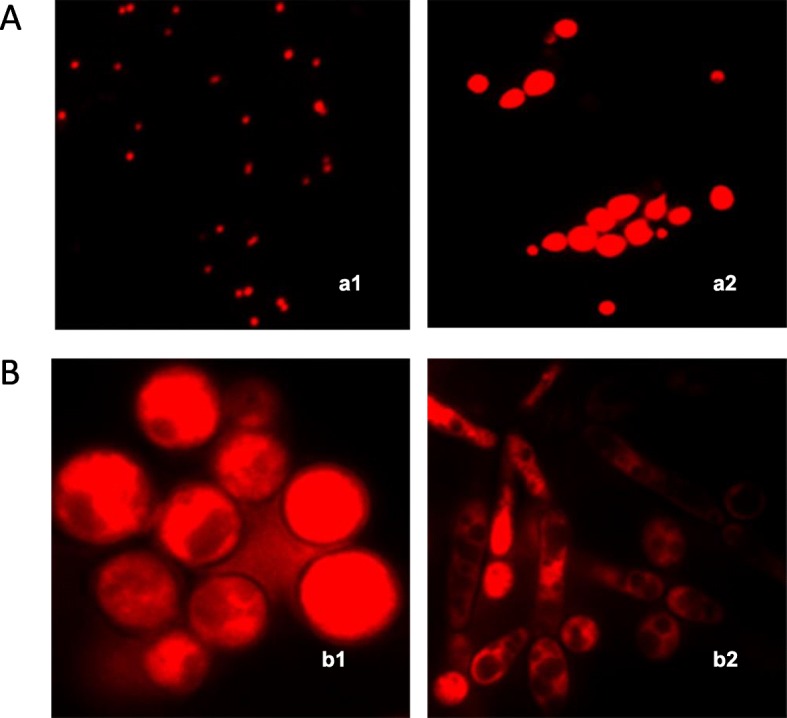
A. Effect of hydrolate on *S. aureus* (a1) and *C. albicans* (a2) by propidium iodide (PI) staining. The images showed PI positive staining of both strains due to altered membrane permeability. B. Effect of hydrolate on *C. albicans* mitocondria by Mitotracker staining. The images showed the mitochondria concentrated in compact masses at one side of the nucleus, none hyphal formation and extracellular material probably released by altered membrane (b1). Mitochondrial morphology of yeast: the images showed punctiform mitochondrial staining in untreated spores and hyphae (b2)

As reported in Table 1, the most abundant components identified in the *C. capitatus* EO were oxygenated structures (70.76%). The largest percentage was represented by phenols, carvacrol (67.58%) and thymol (0.16%), followed by alcoholic monoterpenes, such as β-linalool (0.97%), L-terpinen-4-ol (0.93%), borneol (0.48%) and α-terpineol (0.09%) (Fig. 1). Among the minority oxygenated compounds, a small percentage was represented by alcohols (1-octen-3-ol, 0.21%; 3-octanol, 0.02%), ketones (carvone, 0.04%; camphor, 0.02%; thujone, 0.01%), ethers (eucalyptol, 0.07%), and esters (carvacrol acetate, 0.03%; bornyl acetate 0.01%). Among the not oxygenated compounds (total amount 28.34%), monoterpenes amounted to 19.82% (mainly constituted by γ-terpinene: 6.80%; p-cymene: 6.44%; α-terpinene: 1.88%; β-myrcene: 1.53%; α-pinene: 1.47%; limonene: 0.57%) and sesquiterpenes to 8.50% (β-caryophyllene 7.74%; β-bisabolene: 0.32%; α-bisabolene 0.27%; humulene, 0.14%).

**Table 1:** Constituents of *C. capitatus* EO and hydrolate and their percentage of composition

	EO				Hydrolate		
RT (min)	Peak name	Amount % (v/v)	RI	RT	Peak name	Amount % (v/v)	RI
6.77	Methyl caproate	0.02	884	11.008	Benzyl acohol	0.04	1036
6.882	α-Pinene	1.47	948	12.57	cis-Furan linalool oxide	0.01	1064
7.106	No Match	0.88	n.d.	15.662	Camphor	0.04	1121
7.471	Dehydrosabinene	0.01	957	16.662	endo-Borneol	0.18	1138
7.609	Camphene	0.21	958	17.147	Terpinen-4-ol	0.15	1161
8.494	No Match	0.01	n.d.	17.53	p-Cymen-8-ol	0.02	1172
8.607	β-pinene	0.15	971	17.771	α-Terpineol	0.08	1172
8.687	1-Octen-3-ol	0.21	971	19.495	No Match	0.03	
9.142	β-mircene	1.53	979	22.231	Thymol	6.34	1262
9.321	3-Octanol	0.02	979	22.705	Carvacrol	93.11	1262
9.658	α-Phellandrene	0.33	997				
9.891	trans-β-Ocimene	0.10	1031				
10.142	α-Terpinene	1.88	1033				
10.459	p-Cymene	6.44	1033				
10.637	Limonene	0.57	1039				
10.723	Eucalyptol	0.07	1039				
11.473	α-Ocimene	0.04	1057				
11.896	γ-Terpinene	6.80	1047				
12.246	cis-Sabinene hydrate	0.12	1068				
13.172	Terpinolene	0.14	1078				
13.679	β-Linalool	0.97	1082				
13.909	Thujone	0.01	1096				
14.629	trans-p-2-Menthen-1-ol	0.02	1109				
15.607	Camphor	0.02	1121				
16.573	endo-Borneol	0.48	1138				
17.101	L-Terpinen-4-ol	0.93	1161				
17.724	α-Terpineol	0.09	1172				
20.087	Carvone	0.04	1190				
21.355	α-Citral	0.02	1240				
21.955	Bornyl acetate	0.01	1269				
22.031	No Match	0.01	n.d.				
22.301	Thymol	0.16	1262				
22.782	Carvacrol	67.58	1262				
25.711	Carvacrol acetate	0.03	1421				
27.652	β-Caryophyllene	7.74	1494				
28.45	Alloaromadendrene	0.01	1490				
29.047	Humulene	0.14	1579				
30.748	Ledene	0.02	1520				
31.308	β-Bisabolene	0.32	1500				
32.645	α-Bisabolene	0.27	1521				
34.208	Caryophyllene oxide	0.07	1576				
41.005	No Match	0.01	n.d.				
42.072	Sclarene	0.01	1891				
42.528	Abieta-8 (14),9 (11),12-triene	0.01	2004

The EO content of the hydrolate was gravimetrically evaluated to be 0.1403% (v/v) (Table 1). The main constituents of the hydrolate identified in this study are carvacrol (93.11%) and thymol (6.34%). Terpineols (such as endo-borneol (0.18%), terpinen-4-ol (0.15%), and α-terpineol (0.08%)) and camphor (0.04%) were also present.

The quantitative analysis of carvacrol achieved by interpolation of the GC-MS peak area with the corresponding pure standard calibration curve (y = 119740852.99 x - 1005897752.26,  r2= 0.99946, range of linearity 20–1000 ng) was of 76.31 mg/L (0.0952% v/v), in good accordance with the TGSC information system established for carvacrol water solubility (1250 mg/L) [29].

### Antibacterial activity

Table 2 showed MIC and MBC data for hydrolate and EO against all tested strains. The order of susceptibility to hydrolate (MIC) was *B. subtilis* = *S. aureus* ATCC > *S. aureus* clinical isolates (including MRSA strains) = *S. epidermidis* = *L. monocytogenes* > *P. aeruginosa* strains, with MIC ranged from 12.5 to 50% of product (corresponding to 0.0175–0.07% (v/v) of EO in it). The order of susceptibility to EO (MIC) was *B. subtilis > L. monocytogenes > S. aureus* ATCC > *S. aureus* clinical isolates (including MRSA strains) = *P. aeruginosa* with MIC values ranged from 0.031 to 0.25% (v/v). The MBC values of both hydrolate and EO were generally equivalent or one more concentration above the MIC ones, except for *P. aeruginosa.* The MIC values of TC were in the range from < 0.125 μg/mL to > 4 μg/mL. The order of susceptibility was staphylococci ATCC strains (0.125 μg/mL) > MRSA strains (0.25 μg/mL) = *B. subtilis* ATCC 6633 > *L. monocytogenes* ATCC 13932 (1 μg/mL) > *P. aeruginosa* ATCC 9027 (4 μg/mL) (data not shown).

**Table 2:** MIC and MBC values of hydrolate product of *C. capitatus* compared to EO against bacterial strains

Bacteria	Hydrolate	Hydrolate	EO	EO
	MIC% v/v	MBC% v/v	MIC% v/v	MBC% v/v
*S. aureus* ATCC 6538	12.5 (0.017)^a^	25 (0.035)^a^	0.125	0.125
*S. aureus* 778*6* (c.s.)	25 (0.035)	25 (0.035)	0.25	0.25
*S. aureus* ATCC 43300 (MRSA)	25 (0.035)	25 (0.035)	0.25	0.25
*S. aureus* 815 (MRSA) (c.s.)	25 (0.035)	25 (0.035)	0.25	0.25
*S. aureus* 74CCH (MRSA) (c.s.)	25 (0.035)	25 (0.035)	0.25	0.25
*S. epidermidis* ATCC 34984	25 (0.035)	25 (0.035)	0.25	0.25
*L. monocytogenes* ATCC 13932	25 (0.035)	25 (0.035)	0.062	0.125
*B. subtilis* ATCC 6633	12.5 (0.017)	12.5 (0.017)	0.031	0.062
*P. aeruginosa* ATCC 14552	25 (0.035)	> 50 (> 0.07)	0.125	> 2

*c.s*. Clinical strains

*MRSA* Methicillin resistant *Staphylococcus aureus*

(As essential oil % (v/v) content in hydrolate product)^a^

### Antifungal activity

Table 3 showed MIC and MFC data for hydrolate and EO against all tested yeasts. The order of susceptibility to hydrolate (MIC) was *C. glabrata* > *C. albicans* = *C. guilliermondii* = *C. parapsilosis* > *C. krusei* = *C. tropicalis* > *C. norvegensis* = *C. lusitaniae* = *C. valida* strains*,* with values ranged from 6.25 to 50% of product, corresponding to 0.009–0.07% (v/v) of EO in it*.* The order of susceptibility to EO (MIC) was *C. albicans* ATCC = *C. glabrata* = *C. lusitaniae = C. parapsilosis = C. tropicalis* > *C. krusei = C. albicans* clinical isolate = *C. norvegensis = C. valida* = *C. guilliermondii* strains with MICs from 0.125 to 0.25% (v/v). The MFC values of both hydrolate and EO were generally equivalent or one more concentration above the MIC ones, indicating a fungicidal effect of the two samples. The MIC values of ITC were aligned from 0.5 μg/mL to 8 μg/mL. The order of susceptibility was *C. albicans* ATCC 90028 and clinical strains (0.5 μg/mL) > *C. albicans* ATCC10231 = *C. glabrata* (1 μg/mL) > *C. parapsilosis* = *C. krusei* = *C. lusitaniae* = *C. valida* = *C. norvegensis* = *C. tropicalis* (2 μg/mL) > *C. guilliermondii* (8 μg/mL) (data not shown).

**Table 3:** MIC and MFC values of hydrolate product of *C. capitatus* compared to EO against *Candida* strains

Yeasts	Hydrolate	Hydrolate	EO	EO
	MIC% v/v	MFC% v/v	MIC% v/v	MFC% v/v
*C. albicans* ATCC 90028	12.5 (0.017)^a^	12.5 (0.017)^a^	0.125	0.125
*C. albicans* ATCC 10231	12.5 (0.017)	12.5 (0.017)	0.125	0.125
*C. albicans* 183 (c.s.)	12.5 (0.017)	12.5 (0.017)	0.250	0.250
*C. krusei* 398 (c.s.)	25 (0.035)	25 (0.035)	0.250	0.250
*C. glabrata* ATCC 90030	6.25 (0.009)	12.5 (0.017)	0.125	0.125
*C. glabrata* 32–09 (c.s.)	6.25 (0.009)	25 (0.035)	0.125	0.250
*C. norvegensis* (c.s.)	50 (0.070)	50 (0.070)	0.250	0.250
*C. lusitaniae* (c.s.)	50 (0.070)	50 (0.070)	0.125	0.250
*C. valida* (c.s.)	50 (0.070)	50 (0.070)	0.250	0.250
*C. guillermondii* (c.s.)	12.5 (0.017)	25 (0.035)	0.250	0.250
*C. parapsilosis* (c.s.)	12.5 (0.017)	25 (0.035)	0.125	0.125
*C. tropicalis* (c.s.)	25 (0.035)	50 (0.070)	0.125	0.125

*c.s.* Clinical strains

(As essential oil % (v/v) content in hydrolate product)^a^

### Checkerboard test

The FICIs of hydrolate in association with TC were calculated to ascertain their possible interactions towards all MRSA strains. The combinations of hydrolate with TC showed additive interactions, with FICI values ranged from 0.75 to 1 (Table 4).

**Table 4:** Fractional inhibitory concentration (FIC) and FIC indices (FICI) of antibiotics-hydrolate of *C. capitatus* pairs against MRSA strains

Bacteria	Combination	MICa	MICc	FIC	FICI	Type of interaction
*S. aureus* ATCC 43300	Tetracycline	0.125	0.125	1	1.48	Indifference
	Hydrolate	25 (0.035)^a^	12.5 (0.017)^a^	0.48		
*S. aureus* 815 (MRSA)	Tetracycline	0.25	0.125	0.5	0.98	Additive
	Hydrolate	25 (0.035)	12.5 (0.017)	0.48		
*S. aureus* 74CCH (MRSA)	Tetracycline	0.25	0.125	0.5	0.98	Additive
	Hydrolate	25 (0.035)	12.5 (0.017)	0.48		

(As essential oil % (v/v) content in hydrolate product)^a^

MICa, MIC of one sample alone; MICc, MIC of one sample of the most effective combination

FIC of antibiotic = MIC of antibiotic in combination with hydrolate/MIC of antibiotic alone

FIC of hydrolate = MIC of hydrolate in combination with antibiotic/MIC of hydrolate alone

FICI = FIC of antibiotic+FIC of hydrolate

FICI ≤0.5, synergy; FICI > 0.5 and ≤ 1, additive; FICI > 1 and ≤ 4, indifference; FICI > 4 antagonism

The FICI of hydrolate in combination with ITC was evaluated to determine the possible interactions against *C. albicans*, *C. glabrata* and *C. krusei* clinical strains. The data (Table 5), for hydrolate in combination with ITC, indicated synergistic interaction against *C. krusei* strains (FICI = 0.375), an additive interaction against *C. albicans* strains (FICI = 0.62) and an indifferent interaction against *C. glabrata* strains (FICI = 2). Values of the combination against *C. albicans* and *C. glabrata* indicated indifferent interactions; however, they were considerably lower than the antagonistic value of > 4.

**Table 5:** Fractional inhibitory concentration (FIC) and FIC indices (FICI) of ITC-hydrolate of *C. capitatus* pairs against *Candida* strains

Yeasts		MICa	MICc	FIC	FICI	Type of interaction
*C. kruse*i 398	Itraconazole	2	0.25	0.125	0.375	Synergy
	Hydrolate	25 (0.035)^a^	6.25 (0.009)^a^	0.25		
*C. albicans* 183	Itraconazole	0.5	0.06	0.12	0.62	Additive
	Hydrolate	50 (0.070)	25 (0.035)	0.5		
*C. albicans* ATCC 90028	Itraconazole	0.5	0.125	0.5	1	Additive
	Hydrolate	50 (0.070)	25 (0.035)	0.5		
*C. albicans* ATCC 10231	Itraconazole	1	1	1	2	Indifference
	Hydrolate	50 (0.070)	50 (0.070)	1		
*C. glabrata* 32–09	Itraconazole	1	1	1	2	Indifference
	Hydrolate	6.25 (0.009)	6.25 (0.009)	1		

(As essential oil % (v/v) content in hydrolate product)^a^

MICa, MIC of one sample alone; MICc, MIC of one sample of the most effective combination

FIC of itraconazole = MIC of itraconazole in combination with hydrolate/MIC of itraconazole alone

FIC of hydrolate = MIC of hydrolate in combination with itraconazole/MIC of hydrolate alone

FICI = FIC of itraconazole+FIC of hydrolate

FICI ≤0.5, synergy; FICI > 0.5 and ≤ 1, additive; FICI > 1 and ≤ 4, indifference; FICI > 4 antagonism

### Propidium iodide staining

Propidium iodide, a red-fluorescent nuclear stain, is a membrane impermeant dye that is generally excluded from viable cells. Microscopic examination demonstrated that *S. aureus* ATCC 6538 and *C. albicans* ATCC 10231 cells, treated with hydrolate (0.5 MIC), were stained red (about 84.2–78%, respectively), probably because they lose cell membrane integrity (Fig. 1-Aa1). Moreover, hydrolate clearly inhibited yeast-form growth (Fig. 1-Aa2). The untreated bacterial and yeast cells, about 26.8–21.3% respectively, lost their membrane permeability. Positively stained cells (PI^+^) were observed under inverted fluorescence microscope.

### MitoTracker staining

MitoTracker is a specific mitochondrial stain in live cells and its accumulation depends on the membrane potential. However, once incorporated in the mitochondria, it can chemically link to thiol groups and will not leave the mitochondria when the membrane potential decreases as a result of fixation and/or cell death. Fluorescence microscope images of the treated *C. albicans* ATCC 10231 cells (about 70%) with hydrolate (0.5 MIC) highlighted absence of punctiform mithocondrial staining and showed only diffuse cytoplasmic staining, indicating that mitochondrial function was reduced. Moreover, the staining highlighted morphological changes of treated cells, none hyphal formation and extracellular material between cells caused probably, by the action of the hydrolate on the membrane integrity (Fig. 1-Bb1). The images of healthy non-treated *C. albicans* cells showed punctiform mitochondrial staining (Fig. 1-Bb2).

## Discussion

Antibiotic resistance is a growing public health problem and the discovery and development of new antimicrobial drugs is becoming an important priority [30–32]. In recent years, researchers have been evaluating the antimicrobial activity of many *Thymus* EOs and their components. These compounds are of particular interest as it has never been reported any kind of resistance nor any form of bacterial adaptation to them [33–35].

In this research, we investigated the antimicrobial activity of the hydrolate from *C. capitatus* grown in a very dry territory of southeastern part of the Sicily. This hydrolate contained 0.14% (v/v) of total EO and the carvacrol was the dominant component in it. It exhibited higher antimicrobial activity towards Gram-positive bacteria (including MRSA strains) and yeasts such as *C. glabrata* and *C. krusei*, species that at the present time are often resistant to currently conventional drugs such as azoles (i.e fluconazole) and/or echinocandins (i.e caspofungin) [36]. *C. krusei* is intrinsically resistant to fluconazole and in both these two species the resistance to voriconazole is increasing mainly following exposure to fluconazole [37]. *C. glabrata* is the second most prevalent cause of candidiasis in USA, Australia, and Northern European countries [38]. This yeast has a reduced susceptibility to fluconazole and recently an increase in acquired echinocandin resistance has also been reported [36, 38].

The antimicrobial activity of hydrolate and EO could be related to carvacrol, the main compound contained in both products. Carvacrol (5-isopropyl-2-methylphenol) is a volatile phenolic monoterpene with antimicrobial properties [39, 40]. Terpenes have a great potential to traverse cell walls of bacteria and yeasts due to their large lipophilicity [41, 42]. Their antimicrobial action is due to the hydroxyl group and a delocalized electron system which cause destabilization of the membrane integrity of different microorganisms [1, 43–46]. Hydroxyl groups are highly reactive and form hydrogen bonds with active sites of target enzymes inactivating them and consequently a dysfunction or rupture of the cell membrane [39, 41, 44, 47]. In fact, our findings showed that bacteria and yeasts membrane and mitochondria were affected by hydrolate (Fig. 1 A-B). In addition, carvacrol acts as a proton exchanger, thereby reducing the pH gradient across the cytoplasmic membrane and shows ATPase-inhibiting activity that lead to reduction in other energy-dependent cell processes including synthesis of enzymes and toxins [48, 49]. Furthermore, it inhibits the synthesis of ergosterol in fungi [41].

Other components of this hydrolate, such as thymol and terpinen-4-ol have also demonstrated antimicrobial effects [1, 50]. The synergistic action between carvacrol and these components can occur despite their concentrations were lower than that of carvacrol, suggesting that the hydrolate overall high efficiency is also probably due to its high-water solubility. It has also been found that terpenoids exhibit antiseptic potential according to their solubility in water [51]. Active components of the hydrolate can better diffuse in the aqueous medium around microorganisms and their activity is increased compared to the EO that needs initial solubilisation in an organic solvent (DMSO) before introduction into the aqueous medium [34].

Moreover, the hydrolate in combination with ITC displayed a synergistic effect against *C. krusei* and an additive effect against *C. albicans.* This is a very positive result if it is taken into account the fact that *C. krusei* is intrinsically resistant to fluconazole and can cause breakthrough candidemia in immunocompromised patients receiving long-term prophylactic treatment with this azole. The mechanism of resistance to fluconazole and itraconazole could be due to low sensitivity of the drug target Erg11p to azole antifungals and of the constitutive expression of the multidrug efflux pumps [52].

Whereas in combination with TC, the hydrolate demonstrated an additive effect against MRSA strains. Resistant multi-drug strains, such as MRSA, are becoming a growing worldwide concern and a pressing need to improve MRSA infection therapies has been considered. In fact, *S. aureus* is a major pathogen both in hospitals and in the community [53]. In *S. aureus*, efflux mechanisms are able to confer resistance to various antimicrobial agents including tetracyclines [54].

The hydrolate is able to inhibit microbial growth and to reduce the required active amount of antibiotics when it is in combination. The synergic or additive effects of hydrolate against resistant bacteria and yeasts showed the promising tendency to apply it as an antibiotic adjuvant in combination with drugs. Probably, the main constituent carvacrol after its passage across the cell wall damaged the lipid bilayer of bacterial cell membrane increasing its permeability and enhancing TC effect. The same membrane damage on yeasts enhanced penetration of ITC into the cytoplasm and therefore increased its activity.

## Conclusions

This study reported the characterization of the hydrolate of *C. capitatus* for the first time. It contained traces of total EO and the carvacrol was the main component in it. The antimicrobial activity of hydrolate was mostly direct against resistant bacteria (MRSA strains) and yeasts (*C. glabrata* and *C. krusei*). The mode of action could be due to increase of permeabilization of cell membrane of bacteria and yeasts and mitochondrial dysfunction in yeasts. In addition, the combination of *C. capitatus* hydrolate with antimicrobial agents reduced antibiotic minimum effective dose. Combination therapy between natural compounds and drugs may be able to recover the loss of function for existing antimicrobial agents improving the action and reducing side-effects.

In conclusion, these findings lay the ground for further more extensive investigations in order to identify new natural, cheap, safe and readily available antimicrobial agents with applicability for pharmaceutical topical formulations.

## Data Availability

All data generated or analyzed during this study are included in this published article.

## Abbreviations

CFU: Colony forming unit
DMSO: Dimethyl sulfoxide
ECVs: Epidemiological cut-off values
GC-FID: Gas-chromatography-flame ionization detector
GC-MS: Gas-chromatography-mass spectrometry
ITC: Itraconazole
MIC: Minimum Inhibitory Concentration
MBC: Minimum Bactericidal Concentration
MFC: Minimum Fungicidal Concentration
MHB/MHA: Mueller-Hinton Broth/Agar
MiBat-TUCC: Mycetes and Bacteria/Turin University Culture Collections
PBS: Phosphate buffered solution
TC: Tetracycline
